# Generation of inheritable and “transgene clean” targeted genome-modified rice in later generations using the CRISPR/Cas9 system

**DOI:** 10.1038/srep11491

**Published:** 2015-06-19

**Authors:** Rong-Fang Xu, Hao Li, Rui-Ying Qin, Juan Li, Chun-Hong Qiu, Ya-Chun Yang, Hui Ma, Li Li, Peng-Cheng Wei, Jian-Bo Yang

**Affiliations:** 1 Key Laboratory of Rice Genetic Breeding of Anhui Province, Rice Research Institute, Anhui Academy of Agricultural Sciences, Hefei, 230031, China

## Abstract

The CRISPR/Cas9 system is becoming an important genome editing tool for crop breeding. Although it has been demonstrated that target mutations can be transmitted to the next generation, their inheritance pattern has not yet been fully elucidated. Here, we describe the CRISPR/Cas9-mediated genome editing of four different rice genes with the help of online target-design tools. High-frequency mutagenesis and a large percentage of putative biallelic mutations were observed in T_0_ generations. Nonetheless, our results also indicate that the progeny genotypes of biallelic T_0_ lines are frequently difficult to predict and that the transmission of mutations largely does not conform to classical genetic laws, which suggests that the mutations in T_0_ transgenic rice are mainly somatic mutations. Next, we followed the inheritance pattern of T_1_ plants. Regardless of the presence of the CRISPR/Cas9 transgene, the mutations in T_1_ lines were stably transmitted to later generations, indicating a standard germline transmission pattern. Off-target effects were also evaluated, and our results indicate that with careful target selection, off-target mutations are rare in CRISPR/Cas9-mediated rice gene editing. Taken together, our results indicate the promising production of inheritable and “transgene clean” targeted genome-modified rice in the T_1_ generation using the CRISPR/Cas9 system.

Creating the desired gene diversity in crop plants is the main goal of basic functional genomic research and molecular breeding in agriculture. Gene editing using engineering nucleases, e.g., zinc finger nuclease (ZFN) and transcription activator-like effector nuclease (TALEN), effectively generates genetic variants at specific sites in plant genomes. These nucleases induce site-specific double-strand breaks (DSBs) that are then repaired, leading to genome modifications via homologous recombination (HR) or random deletion/insertion of a small DNA sequence adjacent to the target by non-homologous end-joining (NHEJ). In cooperation with site-specific DSBs, desired genetic elements that are flanked by DNA sequence similarity to the regions of the break point are used to precisely manipulate the target region in genome editing by HR. NHEJ is error-prone and is frequently used to generate deletion/insertion mutations that are likely to cause gene knockout. In plant genome engineering, NHEJ events are more predominant^1^,^2^, but several stable HR-mediated gene-targeting methods have been established for applications in which accuracy is required^3^,^4^. Recently, a more affordable and easier-to-use gene editing system, the clustered regularly interspaced short palindromic repeat (CRISPR)/CRISPR-associated protein 9 (Cas9) system, has evolved from studies of the prokaryote-specific adaptive immune system. In this system, the endonuclease Cas9 is coupled with a guide RNA complex (or a synthetic single guide RNA, sgRNA), generating an RNA-guide nuclease. The specificity of Cas9-directed DNA double-strand cleavage is defined by Watson-Crick base-pairing of a 20-base-pair (bp) guide sequence on the guide RNA (gRNA) and a protospacer-adjacent motif (PAM, “NGG” motif) immediately downstream of the target region. The engineered CRISPR/Cas9 system has been shown to achieve efficient genome editing in a variety of plants, including Arabidopsis, rice, tobacco, wheat, sorghum, maize, tomato, liverwort, and orange^5^,^6^,^7^,^8^,^9^,^10^,^11^,^12^,^13^,^14^,^15^,^16^,^17^,^18^,^19^. Due to its simplicity, rapidness and broad applicability, the CRISPR/Cas9 system is emerging as a powerful tool for various aspects of fundamental studies of plant biology. In addition, the CRISPR/Cas9 system has also been successfully applied to improving traits in crop plants. Through the simultaneous targeting of three copies of a disease resistance locus in the hexaploid genome, a trait for resistance to powdery mildew was artificially created in bread wheat using CRISPR/Cas9 genome editing^20^. Recent work in our laboratory has also demonstrated that an herbicide resistance trait could be rapidly modified in rice through CRISPR/Cas9-mediated gene editing^21^.

Although successful applications of CRISPR/Cas9 to site-specific plant genome editing are accumulating, most data have been collected from transient assays or the first generation of stable transgenic events. Indeed, there are limited studies indicating that the targeted genome modification created by CRISPR/Cas9 can be transmitted in Arabidopsis, tobacco, tomato and rice^5^,^7^,^17^,^22^,^23^,^24^,^25^,^26^,^27^. In Arabidopsis, most mutations in early generations are somatic mutations, leading to difficulty in predetermining the targeted genotype in the next generation^23^,^25^,^28^. In contrast, most putative homozygous mutations generated in rice by a similar CRISPR/Cas9 system have been suggested to be germline mutations, which can be transmitted according to classical inheritance laws^26^. Because the editing achieved might vary among different species, target sites, transgene methods and constructions of the CRISPR/Cas9 complex, the transmission patterns that occur still require further exploration in crops. In addition, although inheritance patterns have been intensively examined in later Arabidopsis generations (T_2_ to T_3_)^12^,^24^,^25^, there are only preliminary data showing the genetic transmission pattern of modifications in later generations (T_1_ to T_2_) of rice^27^. Therefore, it is worth investigating the inheritance of targeted editing in later rice generations in more detail. Off-target effects are another major concern in the application of the CRISPR/Cas9 system. The 20-bp gRNA sequence determines the specificity of CRISPR/Cas9, and a “seed region” of 6–12 bp immediately upstream of the PAM is most essential for the stringency of target recognition^29^. Off-target mutations were indeed induced by CRISPR/Cas9 in human cells^29^,^30^,^31^, though the specificity of CRISPR/Cas9 in plants remains unclear. Independent studies using in-depth whole-genome sequencing and large-scale screening suggest that off-target mutations are rare in Arabidopsis, rice and tobacco^12^,^13^,^17^,^26^. However, a relatively high frequency of off-target mutations was observed while generating the multi-gene knockout of a rice gene family^32^. Although there are two mismatches in the 20-bp gRNA region, including one mismatch in the potentially conserved “seed region”, off-target mutations still show comparable efficiencies with that of on-target editing^32^. The careful design of gRNAs has been suggested to be effective in avoiding off-target mutations in animal cases^31^,^33^, and several bioinformatic tools have been developed to facilitate sgRNA design in plants^34^,^35^,^36^, though their stability has not been experimentally tested.

In this study, four rice genes were targeted using computationally designed gRNA with the stably transformed CRISPR/Cas9 system. Targeted mutagenesis was examined in T_0_ and later generations to determine the transmission pattern of the genome editing achieved. Off-target effects were also evaluated. Our results suggest that the inheritable and “transgene clean” targeted genome modification of rice in the T_1_ generation can be generated by the CRISPR/Cas9 system.

## Results

### CRISPR/Cas9-mediated targeted mutagenesis in T_0_ transgenic rice

The rice *AOX1* family is composed of three members (*OsAOX1a*, *OsAOX1b* and *OsAOX1c*) with high sequence similarity. To introduce individual mutations, we designed specific 20-bp gRNAs with at least a two-base mismatch at potential off-target sites using bioinformatic tools^34^,^35^. These gRNAs were inserted into a GATEWAY-based vector system using a rice-codon-optimized *Cas9* gene and an OsU3 promoter, as previously reported^7^. To evaluate the off-target effects of the system, a 20-bp region of a P450 gene, *OsBEL*, was selected and constructed. This region has similarity to a sequence located ~7 kb upstream of Os*BEL*, with a one base mismatch 13 bp upstream of the PAM.

Through *Agrobacterium*-mediated stable transformation in Nipponbare, 8, 7, 12 and 14 independent T_0_ transgenic events were obtained, carrying constructs targeting *OsAOX1a*, *OsAOX1b*, *OsAOX1c* and *OsBEL*, respectively. To detect mutations, genomic DNA was isolated from the third leaf from the top of 10-week-old plants. The target regions were analyzed by sequencing the products of the corresponding site-specific genomic PCR and/or further confirmed by sequencing clones of the PCR amplicons. High mutation rates were induced in all tested targets, and more than half of the lines of each transgene carried mutations (Table 1, Supplemental Fig. S1-S4). The highest mutagenesis efficiency was observed at the *OsBEL* site, in which the target region was modified in 12 lines out of a total of 14 lines (Table 1, Supplemental Fig. S4). To investigate the possible reason for unsuccessful target mutagenesis, the presence of the transgene was determined by amplifying *sgRNA*, *Cas9*, and *hygromycin phosphotransferase* (*HPT*) in non-mutated lines (designated as WT). Among a total of 12 WT lines, 6 lines lacked detectable *Cas9* and/or *sgRNA* transgene fragments (Supplemental Table S1), implying that a deficiency in the integrity of the sgRNA/Cas9 expression cassette might be a major reason for the failure of targeted mutagenesis. We also tested the numbers of T-DNA insertions in all transgenic lines by determining the copy number of *HPT* via real-time PCR analysis. Most lines carrying target mutations contained 1–2 copies (Table 1), suggesting that it may be possible to segregate out the transgene.

Some reports have indicated that 1-bp changes (deletions or insertions) are the main type of mutation induced by stably transformed CRISPR/Cas9 in both Arabidopsis and rice^23^,^26^. However, a remarkable abundance of deletions of long fragments (≥ 3 bp) were observed in a study of high-efficiency gene editing targeting rice *SWEET13*^27^. We found that the types of mutations varied among target regions. For *OsAOX1c* and Os*BEL*, short changes (≤ 3 bp) were the major type of mutation; conversely, all mutations in *OsAOX1a* were relatively long deletions. Because short deletions are often associated with classical NHEJ (cNHEJ) and longer deletions may represent the results of microhomology-mediated end-joining (or alternative NHEJ, aNHEJ)^37^,^38^, the CRISPR/Cas9-induced mutation patterns might be different for specific DSB sites through distinguished NHEJ repair pathway.

The genotypes of the mutants were also analyzed. Consistent with previous reports^26^,^27^, putative biallelic i.e., homozygous or compound heterozygous mutations mostly occurred in genotypes in the T_0_ generation, accounting for 41.4% (12/29) and 31.0% (9/29) of all mutant plants, respectively.

### Inheritance and stability of targeted mutagenesis in the T_1_ generation

To investigate the pattern of transmission of CRISPR/Cas9-mediated targeted gene modification, several T_1_ progeny were obtained by strict self-pollination and used for testing of targeted mutations. For each T_0_ line, 8-24 progeny were randomly selected and examined. As shown in Table 2, all of the mutated T_0_ lines produced mutated T_1_ progeny, whereas targeted sequence changes still could not be detected in the progeny of WT T_0_ plants.

Genotypes are thought to be easily predicted in the progeny of biallelic T_0_ lines^26^. As expected, all 12 and 16 T_1_ progeny of homozygous *OsBEL* #1 and #3, respectively, exhibited consistent homozygous 12-bp or 1-bp deletion genotypes (Table 2). However, we observed that the transmission of targeted mutations was relatively ruleless in large subsets of putative biallelic T_0_ lines. Three unexpected patterns can be summarized as follows. (1) The mutations occurring in T_0_ were lost in the T_1_ generation. *OsAOX1a* line #1 was determined to be a putative biallelic mutation with 7-bp and 11-bp deletions, whereas only the 11-bp deletion could be detected in T_1_ plants as a homozygous genotype (Table 2). In *OsAOX1a* #2 and *OsAOX1b* #2 and #3, the selective transmission of a single parental allele was also found in the progeny. (2) New mutations were created in the T_1_ generation (Table 2, Supplemental Fig. S5). For example, the sequencing results indicated a putative homozygous 1-bp insertion genotype in an *OsAOX1c* #4 T_0_ plant, whereas 2 additional different 1-bp insertions were found in the T_1_ population (Table 2). Similarly, a number of additional mutations were detected in the T_1_ generations of *OsAOX1c* #9 and *OsBEL* #2, even though the majority of progeny mutations were already observed in the parental genome. In addition, several putative biallelic mutated T_0_ lines, e.g., *OsAOX1c* #3, #9 and *OsBEL* #2, could generate progeny carrying the WT allele. This result suggested that some cells of the T_0_ plants might not be target modified in these lines. Meanwhile, these cells were also not detected in the genotyping of T_0_ plants. (3) The segregation ratio of target mutations in the T_1_ generation was distorted. A 1:1 ratio of the parental mutated alleles was anticipated in the progeny of biallelic plants, based on regular segregation laws. In the T_1_ plants of the biallelic mutated *OsAOX1b* #1 lines, although the mutation types did not increase or decrease, the ratio between the two alleles in the T_1_ plants did not conform to 1:1, indicating that they were not inherited with equal frequencies.

Because the sgRNA-Cas9 complex has been shown to be active in heterozygous and chimeric plants^5^,^24^,^26^,^27^, WT alleles are likely to be modified continuously. As expected, a number of new mutations were found in the corresponding T_1_ lines (e.g., *OsAOX1c* #6 and #7; *OsBEL* #7 and #8), whereas most of the mutations detected in the T_0_ heterozygotes and chimeras were passed on to the next generation (Table 2). Whether or not there was additional mutation of T_0_ heterozygotes in the T_1_ generation, the ratio between the parental mutated allele and other alleles should be expected to be 1:1 in the T_1_ population. However, the frequency of the parental mutation in some T_1_ generated from T_0_ heterozygotes, e.g., *OsAOX1c* #6 and *OsBEL* #6, was significantly lower than 50% by the chi-square test, suggesting that additional mutations likely occurred in other undetermined parts of the T_0_ plants.

The presence of the transgene (T-DNA) region was also examined in T_1_ populations. The absence of the transgene was determined to be concurrent in PCRs negative for *Cas9*, sgRNA and *HPT* genes, and the results indicated that the T-DNA region could be segregated out in most lines. Transgene-negative plants were observed in nearly all of the low-copy T_0_ progeny (Table 2).

### Segregation of targeted mutagenesis in T_2_ generations

As described above, intricate segregation patterns were detected in the T_1_ generation. To further investigate the inheritance of targeted mutations in later generations, the genotypes of several T_2_ plants were analyzed in detail. Because the sgRNA/Cas9 complex may still be active in progeny and thus disturb genotype transmission, the segregation of mutations in T-DNA-lacking T_1_ progenitors was examined first. A total of 12 T_1_ lines carrying 3 genotypes (8 homozygous, 3 compound heterozygous and 1 heterozygous) and lacking the transgene were selected and analyzed. By sequencing targeted genomic regions of an extensive T_2_ population derived from T_1_ homozygotes, all of the descendants were found to exhibit the same homozygous mutations, without exception (Table 3). Similarly, the ratio between the two alleles of the biallelic and heterozygous T_1_ plants conformed to the expected 1:1 ratio of classical Mendelian segregation by the chi-square test (Table 3). All of these results indicate that, in the absence of the transgene, the inheritance of targeted mutations is stable and regular in later generations. Furthermore, the patterns of transmission from T_1_ to T_2_ were examined in the presence of the transgene. For this assay, 4 T-DNA-positive T_1_ homozygous and 2 compound heterozygous T_1_ lines were selected, and the genotypes were examined in their progeny. As shown in Table 4, the parental mutations were not modified or revised in the T_2_ generation, possibly due to the absence of editable targets of CRISPR/Cas9. We also followed 4 T_1_ heterozygotes to the T_2_ generation and found that most of the genotypes were inherited normally, with only one additional mutation detected in a single T_2_ plant (Table 4).

### Off-target analysis

Based on the predictions of the CRISPR-P tool, we first analyzed the off-target effects of the editing of *OsAOX1* genes. The two most likely off-target sites of each target were selected and examined in all of the T_0_ plants, all of the T-DNA-negative T_1_ plants generated from mutated T_0_ lines and 24 randomly selected lines of T-DNA-positive T_1_ plants with on-target mutation by site-specific genomic PCR based Sanger sequencing. As shown in Table 5, no mutations were found in the putative loci, even though on-target mutations could easily be detected.

According to previous reports and bioinformatic tools, the editing of *OsBEL* is very likely to be an off-target event because the selected 20-bp gRNA region is highly homologous (1 bp mismatched outside of the seed region) with the other PAM ended sequence. The targeted site and the putative off-target site are 7 kb apart; therefore, we first examined the large deletion formed by the re-joining of the two cleavage sites. We did not detect a deletion between the two sites by PCR in any of the T_0_ and T_1_ generation *OsBEL* target plants (data not shown). To further evaluate the potential off-target effects of the CRISPR/Cas9 system in rice, we used PCR to amplify a 254-bp region around the putative site and then sequenced that region. It was not changed in any of the T_0_ and transgene-negative T_1_ plants. We further examined 60 lines of transgene-positive T_1_ plants, and mutations were observed at off-target sites in two individual plants derived from different T_0_ lines (Table 5). These results suggest that in this system, off-target modifications are rare and occur only in the transgene-positive T_1_ generation.

## Discussion

**Figure 1:**
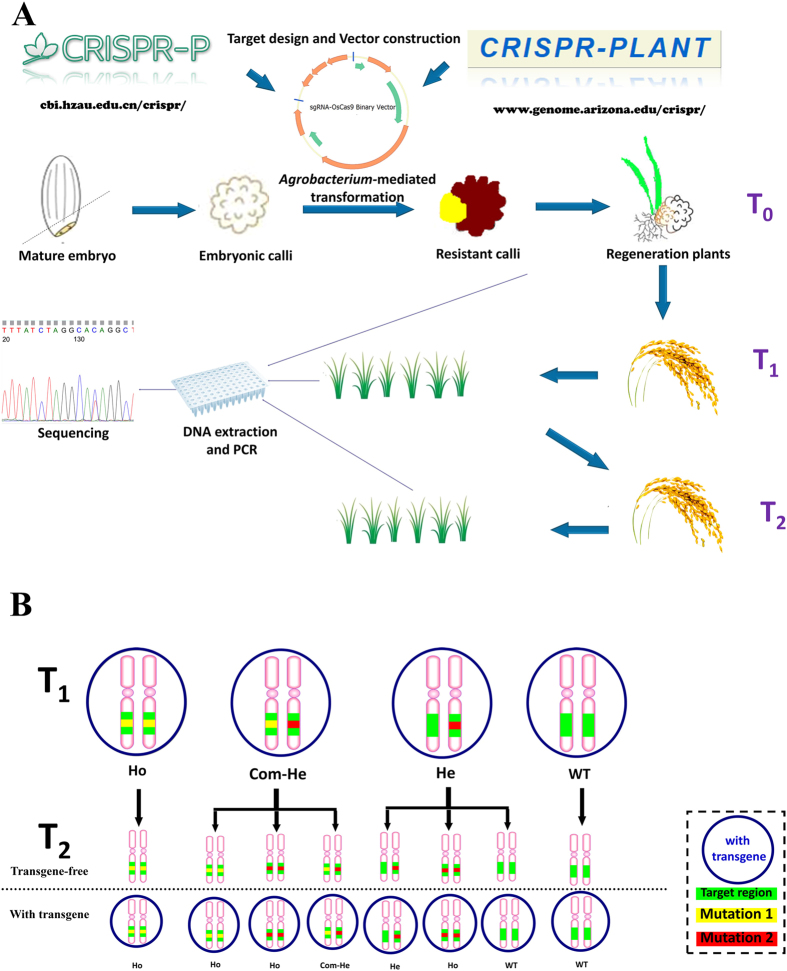
Production of rice plants with inheritable desired mutations. **A**, Schematic of the procedure for the generation and analysis of targeted mutated plants. The target site was selected using CRISPR-P or CRISPR-Plant tools and inserted into a binary vector to express sgRNA and Cas9. T_0_ plants were regenerated by *Agrobacterium*-mediated transformation, and later generations were produced by strict self-pollination. In each generation, the targeted mutations were examined by site-specific PCR and sequential sequencing. **B**, The overview of the major inheritance patterns of transgene-positive T_1_ plants. A circle indicates that the plant carries the transgene in the genome. Green indicates the targeted regions. Yellow and red indicate different mutations on the target site. The indicated zygosity of homozygote (Ho), compound heterozygote (Com-He) and heterozygote (He) is putative.

The predictable inheritance and segregation of genome modifications in later generations is highly desired in molecular breeding as well as in basic research. In this study, we targeted four different genes using a previously reported Gateway-based CRISPR/Cas9 system^7^. The schematic procedure of generation and analysis of targeted mutated plants was described in Fig. 1A. Our results confirm the high efficiency of this system in the T_0_ generation. We found that a part of the un-mutated lines lacked the sgRNA, the Cas9 cassette, or even both, which is consistent with another rice CRISPR/Cas9 application using a different vector system^27^. Interestingly, the left border (LB) of T-DNA is easier to truncate during the integration. However, the selectable marker was located closer to LB than the transgene fragments of CRISPR/Cas9^39^. These phenomena suggest that the possible recombination of the T-DNA fragment might be a potential reason to restrict the mutagenesis efficiency. According to the sequencing results for genomic DNA isolated from single leaves, a large percentage of edited T_0_ generations are biallelically modified. An abundance of biallelic modifications, especially homozygous types, typically indicates that the mutations were generated at a very early developmental stage of the transformed embryogenic cell, suggesting the high possibility of predictable germline transmission^26^. However, we found that T_1_ genotypes are not easily predicted. Increases and decreases in types of mutation were frequently found not only in heterozygous and chimeric lines but also in putative biallelic lines, and the segregations were distorted even when the mutation type was stably transmitted. Various lines, e.g., *OsAXO1a* #2, *OsAOX1b* #3, *OsAOX1c* #4 and *OsBEL* #2, showed that the T-DNA region was segregating according to standard laws by the chi-square test, whereas the transmission of targeted mutations was disrupted (Table 2). One possible explanation is that the abnormal inheritance was caused by somatic mutations. For example, putative biallelic plants are actually chimeras with different homozygous mutations in separate cells. The mutations thus may have been lost or inherited unequally during germline segregation. Meanwhile, the PCR-based detection method has limitations in the detection of larger deletions because the PCR reaction would fail if the deletion removed the primer-binding sites. Therefore, the mutation frequency might be underestimated, and the failure of detection of the mutated allele might confound the analysis of inheritance patterns in certain lines. Moreover, it has been reported that different mutations can be detected in samples of different tissues^26^. Because we only examined the target sequence in a single leaf, it would not be surprising that some genotypes present in the rest of the plant were overlooked. Therefore, the additional mutations found in T_1_ might be transmitted from undetected T_0_ somatic mutations. Unexpected inheritance patterns of T_0_ plants have also been observed in other studies, but with lower frequency^26^,^27^. Although there are no significant differences in the mutation rates of the two CRISPR/Cas9 systems^7^,^10^, the differences in the system construction still might be a reason for the observed variation in the frequency of somatic mutations. Compared to the random and complicated genetic transmission in the first generation, the patterns are stable and easier to predict in later generations. Except for newly occurring mutations, all of the T_2_ genotypes were inherited normally from T_1_ plants in the presence or in the absence of the transgene (Fig. 1B), showing standard germline transmission.

Off-target events are an important concern in the application of CRISPR/Cas9 in plants. A double nicking approach, combining paired sgRNAs with distinct locations adjacent to the target site and a nickase version of mutated Cas9, was reported to effectively avoid off-target effects^28^,^40^, but it would limit the potential target range compared to the single sgRNA/Cas9 system. The off-target efficiency may vary greatly depending on the construction of CRISPR/Cas9, the organism and the transformation method. For the four different targets in this study, we found low-frequency mutations in only one off-target site, which had a 1-bp mismatch outside of the seed region, with on-target sites in the T_1_ generation plant in the presence of the transgene. These results indicate that the off-target effect is indeed quite low in rice targeted gene modification using the vector and transformation system described here. By selecting target sites with the help of bioinformatic tools^34^,^35^, we successfully generated mutations in three individual *AOX1* family members, which is difficult to achieve using standard RNAi methods (data not shown) due to the high sequence similarity. These results demonstrate the reliability of software-aided target selection methods and also suggest that the off-target events in the highly efficient CRISPR/Cas9 system could be virtually avoided in rice-gene editing through careful sgRNA design.

In animals, biallelic mutations can be efficiently generated in one-cell-stage embryos by microinjecting an excessive amount of sgRNA and *Cas9* RNA^41^,^42^,^43^. This method nearly guarantees reliable germline transmission both in theory and in practice. However, the integration of the CRISPR/Cas9 sequence into the genome is necessary for generating targeted modifications in plants. *Agrobacterium*-mediated transformation of embryogenic calli is a common method for generating transgenic crops. The transformed cells soon divide, allowing only a short time window for generating the germline mutation. In contrast, the regeneration of transgenic crop plants from embryogenic cells normally requires several weeks or months, and the sgRNA/Cas9 complex should be continuously expressed during this period. This long expression period may give rise to the high risk of somatic mutations in the first generation. The intricate T_0_ segregation pattern in this report strongly supports the widespread occurrence of somatic mutations. In contrast, we revealed that T_1_ mutations, especially biallelic mutations, were stably transmitted to the next generation through germline transmission. An advantage of the CRISPR/Cas9-mediated gene editing is the potential for transgene-free progeny. Once the desired gene editing has been achieved, the transgene region can be easily segregated out in progeny via simple self-fertilization. In this study, we show that the T-DNA had indeed completely segregated out in the T_1_ generation, with the targeted mutation transmitting independently. In addition, our results reveal that off-target mutations were only found in the transgene-positive T_1_ plants. Therefore, the off-target effects might be largely reduced by selecting appropriate T_1_ progeny. Taken together, our results indicate that stable inheritance and “transgene clean” homozygous targeted gene editing can be produced in the T_1_ generation in CRISPR/Cas9-transgenic rice. Therefore, the system can be used as a simple, rapid and powerful molecular tool in crop variety improvement and will greatly advance molecular design in breeding.

## Materials and Methods

### Plant material and growth conditions

Rice plants (*Oryza sativa* L. ssp. *japonica*) were used for plant transformation. Mature, non-dormant seeds were sterilized and germinated in 1/2 MS medium under a light/dark cycle of 16 h/8 h at 28 °C for at least 10 days. Rice seedlings at the trifoliate stage or regenerated rice after 4 weeks of rooting were transferred to plastic buckets in a greenhouse maintained at 30 °C during the day and 28 °C at night.

### Vector construction and rice transformation

The oligonucleotides used for targeted mutagenesis were designed with the help of the CRISPR-P and CRISPR-PLANT tools^34^,^35^ and are listed in Supplemental Table S2. The Gateway-based CRISPR/Cas9 plant expression vectors were constructed as previously described^7^. The binary constructs were then introduced into the *Agrobacterium tumefaciens* strain EHA105. Embryonic calli from mature rice seeds were transformed by co-cultivation, selected with 50 mg/l hygromycin, and used to regenerate transgenic plants as previously described^44^. The numbers of transgene copies were determined using real-time PCR^45^.

### Genotyping

Total DNA was extracted and purified from approximately 100 mg mature rice leaves followed by a previously described high-throughput method^46^. Specific PCR primers, as listed in Supplemental Table S2, were used to examine the presence of T-DNA regions. To detect mutations, the genomic regions surrounding on- and off- target sites were amplified using specific PCR primers. The fragments were directly sequenced using the corresponding site-specific primers or cloned into the pEASY-T vector and then Sanger-sequenced using the M13 primer.

## Additional Information

**How to cite this article**: Xu, R.-F. *et al.* Generation of inheritable and "transgene clean" targeted genome-modified rice in later generations using the CRISPR/Cas9 system. *Sci. Rep.* **5**, 11491; doi: 10.1038/srep11491 (2015).

## Supplementary Material

Supplementary Information

## Figures and Tables

**Table 1:** Identification of CRISPR/Cas9-induced target mutations in T_0_ generations.

Target Gene	Line#	Genotype*	Zygosity#	Copy Number
*OsAox1a*	1	4d2d5,5d11a	Com-He	2
	2	7d44,3d29	Com-He	1
	3	8d11b	Ho	1
	4	2d3,5d6	Com-He	1
	5	9WT	WT	1
	6	WT	WT	2
	7	WT	WT	1
	8	WT	WT	≥3
*OsAox1b*	1	9d2,3d4	Com-He	1
	2	4d3,4d69	Com-He	2
	3	6d1,1d31	Com-He	1
	4	9d1,2d4	Com-He	1
	5	WT	WT	1
	6	WT	WT	2
	7	WT	WT	1
*OsAox1c*	1	8d2	Ho	1
	2	4d2	Ho	≥3
	3	7i1a	Ho	1
	4	9i1b	Ho	2
	5	5s1,4WT	He	2
	6	8i1c,3WT	He	1
	7	6d10,5WT	He	1
	8	7i1b,3s1	Com-He	1
	9	5d5,3d3	Com-He	1
	10	WT	WT	2
	11	6WT	WT	1
	12	WT	WT	≥3
*OsBEL*	1	10d12	Ho	≥3
	2	11d4	Ho	2
	3	9d1	Ho	1
	4	12d1	Ho	1
	5	5d1,5WT	He	2
	6	4d6,3WT	He	2
	7	6d4,6WT	He	1
	8	2d1,2d2,2d3a,4WT	Ch	2
	9	3d1,5d2,3WT	Ch	1
	10	7d1,3i1	Com-He	2
	11	8d1,2d4	Com-He	2
	12	5d1,5d8	Com-He	1
	13	WT	WT	1
	14	WT	WT	1

^*^WT, wild-type sequence with no mutation detected; d#, # of bp deleted from the target site; d#a, the same number of deletions at one site; d#b, the same number of deletions at other sites; s#, # of bp substituted from the target site. i#, # of bp inserted at the target site; i#a, the same number of insertions at one site; i#b, the same number of insertions at other sites; i#c, the same number of insertions at the third site.

^#^The zygosity of homozygote (Ho), compound heterozygote (Com-He), heterozygote (He) and chimera (Ch) in T_0_ plants is putative.

**Table 2:** Segregation patterns of CRISPR/Cas9-transgenic plants during the T_0_ to T_1_ generation.

Line	T_0_		T_1_Segregation ratio	
	Genotype	Zygosity*	Targeted mutation#	T-DNA$
*OsAOX1a*#1	d2d5,d11a	Com-He	24d11ad11a	23+:1−
*OsAOX1a*#2	d44,d29	Com-He	17d44d44	13+:4−
*OsAOX1a*#3	d11bd11b	Ho	20d11bd11b:3He:1d8d11b	20+:4−
*OsAOX1a*#4	d3,d6	Com-He	8d6d6: 3d3d6:13He	15+:9−
*OsAOX1a*#7	WT	WT	24WT	18+:6−
*OsAOX1b*#1	d2,d4	Com-He	13d2d2:7d2d4:4d4d4	17+:7−
*OsAOX1b*#2	d3,d69	Com-He	19d69d69	16+:3−
*OsAOX1b*#3	d1,d31	Com-He	24d31d31	19+:5−
*OsAOX1b*#4	d1,d4	Com-He	2d1d1: 6d1d4:3d4d4	8+:3−
*OsAOX1b*#5	WT	WT	16WT	10+:6−
*OsAOX1b*#6	WT	WT	18WT	18+
*OsAOX1c*#1	d2d2	Ho	5d1d1:3d2d2:14d1d2	16+:6−
*OsAOX1c*#3	i1ai1a	Ho	6i1ai1a:4He	7+:3−
*OsAOX1c*#4	i1bi1b	Ho	2i1ai1a:3i1bi1b:1i1ci1c:4He:7WT	16+:1−
*OsAOX1c*#6	i1c,WT	He	6i1ci1c:3i1bi1c:5He#1:2He#2:3WT	13+:6−
*OsAOX1c*#7	d10,WT	He	2d10d10:1d10d52:3He#1:3He#2:5WT	9+:5−
*OsAOX1c*#9	d5,1d3	Com-He	1d5d5:5ch:10He:2WT	15+:3−
*OsAOX1c*#12	WT	WT	19WT	19+
*OsBEL*#1	d12d12	Ho	12d12d12	12+
*OsBEL*#2	d4d4	Ho	5d4d4:4d4d1:2d4d25:6He	13+:4−
*OsBEL*#3	d1d1	Ho	16d1d1	15+:1−
*OsBEL*#6	d6,WT	He	5d6d6:3He:16WT	20+:4−
*OsBEL*#7	d4,WT	He	7d4d4:2d4d3b:5d1d1:4He:1Ch	14+:5−
*OsBEL*#8	d1,d2,d3a,WT	Ch	2d1d1:5d1s1d1s1:1Ch	7+:1−

^*^The zygosity of homozygote (Ho), compound heterozygote (Com-He), heterozygote (He) and chimera (Ch) in T_0_ plants is putative.

^#^The genotypes in the T_1_ generation were as follows: *OsAOX1a*#3 He (d11b, WT); *OsAOX1a*#4 He (d3, WT); *OsAOX1c*#3 He (i1a, WT); *OsAOX1c*#4 He (i1b, WT); *OsAOX1c*#6 He#1 (i1c, WT); *OsAOX1c*#6 He#2 (i1b, WT); *OsAOX1c*#7 He#1 (d10, WT); *OsAOX1c*#7 He#2 (d52, WT); *OsAOX1c*#9 He (d5, WT); *OsAOX1c*#9 Ch (d2, d3, d5); *OsBEL*#2 He (d4, WT); *OsBEL*#6 He (d6, WT); *OsBEL*#7 He (d1, WT); *OsBEL*#7 Ch (d1, d3b, d20); *OsBEL*#8 Ch (d1, d4, d45, WT).

^$^+, The number of T-DNA regions that were detected; -, the number of T-DNA regions that were not detected.

**Table 3:** Segregation patterns of CRISPR/Cas9 modifications during the T_1_ to T_2_ generation in the absence of the transgene region.

Line		T_1_			T_2_	
	Genotype	Zygosity	T-DNA	Segregation ratio*	T-DNA#	
*OsAOX1a* #1–3	d11ad11a	Ho	—	24d11ad11a	24—
*OsAOX1a* #2–2	d44d44	Ho	—	21d44d44	21—
*OsAOX1a* #2–17	d44d44	Ho	—	24d44d44	24—
*OsAOX1a* #3–4	d11bd11b	Ho	—	18d11bd11b	18—
*OsAOX1b* #1–1	d4d4	Ho	—	20d4d4	20—
*OsAOX1b* #2–9	d69d69	Ho	—	22d69d69	22—
*OsAOX1b* #3–4	d31d31	Ho	—	24d31d31	24—
*OsAOX1b* #4–7	d1d1	Ho	—	20d1d1	20—
*OsAOX1a* #3–8	d8,d11b	Com-He	—	5d8d8:13d8d11b:4d11bd11b	22—
*OsAOX1a* #4–3	d3,d6	Com-He	—	7d3d3:11d3d6:6d6d6	24—
*OsAOX1b* #4–5	d1,d4	Com-He	—	3d1d1:8d1d4:6d4d4	17—
*OsAOX1a* #4–12	d3,WT	He	—	5d3d3:11He:7WT	23—

^*^The genotype of the**

OsAOX1a#4-12 He plant in the T_2_ generation was (d3, WT).

**Table 4:** Segregation patterns of CRISPR/Cas9 modifications during the T_1_ to T_2_ generation in the presence of the transgene region.

Line	T_1_			T_2_ Segregation ratio*
	Genotype	Zygosity	T-DNA	
*OsAOX1a* #1–1	d11ad11a	Ho	+	12d11ad11a
*OsAOX1a* #4–9	d6d6	Ho	+	12d6d6
*OsAOX1b* #2–3	d69d69	Ho	+	10d69d69
*OsAOX1b* #4–13	d1d1	Ho	+	12d1d1
*OsAOX1a* #4–19	d3d6	Com-He	+	2d3d3:5d3d6:3d6d6
*OsAOX1b* #4–2	d1d4	Com-He	+	1d1d1:8d1d4:3d4d4
*OsAOX1a* #3–14	d11b,WT	He	+	2d11bd11b:7He:3WT
*OsAOX1a* #4–1	d3,WT	He	+	1d3d3:5He:1Ch:2WT
*OsAOX1a* #4–2	d3,WT	He	+	3d3d3:4He:4WT
*OsAOX1a* #4–18	d3,WT	He	+	5d3d3:3He:2WT

^*^The genotypes in the T_2_ generation were as follows: *OsAOX1a*#3–14 He (d11b, WT); *OsAOX1a*#4–1 He (d3, WT); *OsAOX1a*#4–1 Ch (d3, d5, WT); *OsAOX1a*#4–2 He (d3, WT); *OsAOX1a*#4–18 He (d3, WT).

**Table 5:** Detection of mutations on the putative off-target sites.

Target	Name of putative off-target site	Putative off-target locus	Sequence of the putative off-target site	No. of mismatching bases	*No. of plants sequenced	No. of plants with mutations
*OsAOX1a*	OFF1	Chr6:8886912-8886890	GAGTCGTGGTCAACAGCTAGGGG	4	50	0
	OFF2	Chr6:13427405-13427383	AGGTGGTGGCCACCAGCTCCTGG	3	50	0
*OsAOX1b*	OFF3	Chr8:22659044-22659066	CAGCGAGGTGAGCTCGCGAAAGG	3	49	0
	OFF4	Chr11:6535738-6535715	CTCAGCGATGAGCTCCTGAAGGG	4	49	0
*OsAOX1c*	OFF5	Chr10:20144706-20144728	GGAGGAGGCGGCCGCGTCCTCGG	2	60	0
	OFF6	Chr2:15363651-15363629	GGCGGAGGCGGCCGCGTCCTGGG	3	60	0
*OsBEL*	OFF7	Chr3:31436831-31436853	GCGAGGTGCGCGCCATGGTGCGG	1	89	2
	OFF8	Chr4:23949393-23949415	GAGAGGTGGGCGCCATGGTGGGG	3	89	0

The PAM motif (NGG) is marked by a box; mismatching bases are shown in red.

^*^For targets of *OsAOX1a*, *OsAOX1b* and *OsAOX1c*, all of the T_0_ plants, all of the T-DNA-negative T_1_ plants generated from mutated T_0_ lines, and 24 randomly selected lines of T-DNA-positive T_1_ plants with on-target mutation were used. For *OsBEL* targets, all of the T_0_ plants, all of the T-DNA-negative T_1_ plants generated from mutated T_0_ lines, and 60 randomly selected lines of T-DNA-positive T_1_ plants with on-target mutation were used.
