# Supplementary material for: Generation of inheritable and “transgene clean” targeted genome-modified rice in later generations using the CRISPR/Cas9 system
Source: Sci Rep. 2015 Jun 19;5:11491. doi: 10.1038/srep11491 (PMC5155577; doi:10.1038/srep11491)
Supplement: Supplementary Information [file srep11491-s1.pdf]

Supplementary Information

Generation of inheritable and "transgene clean" targeted genome-modified rice in later generations using the CRISPR/Cas9 system.

Rong-Fang Xu, Hao Li, Rui-Ying Qin, Juan Li, Chun-Hong Qiu, Ya-Chun Yang, Hui Ma, Li Li, Peng-Cheng Wei & Jian-Bo Yang

Supplemental Figure S1

A *OsAOX1a* site

	CGGAGAAGGAGGTGGTGGTCAACAGCTACTGGGGCATCG	WT
Plant ID		
Wild-type	CGGAGAAGGAGGTGGTGGTCAACAGCTACTGGGGCATCG	
#1	CGGAGAAGGAGGTGGTGGTC----C----------CTGGGGCATCG	d2d5(x4)
	CGGAGAAGGAGGTGGT----------------------ACTGGGGCATCG	d11a(x5)
#2	CGGAGAAGGAGGTGGTGG--…	d29(x3)
	CGGAGAAGGAGGTGGTGG--…	d44(x7)
#3	CGGAGAAGGAGGTGGTGGTCAACAG----------------------TCG	d11b(x8)
#4	CGGAGAAGGAGGTGGTGGTCAAC------TACTGGGGCATCG	d3(x2)
	CGGAGAAGGAGGTGGTGGTC------------TACTGGGGCATCG	d6(x5)

B

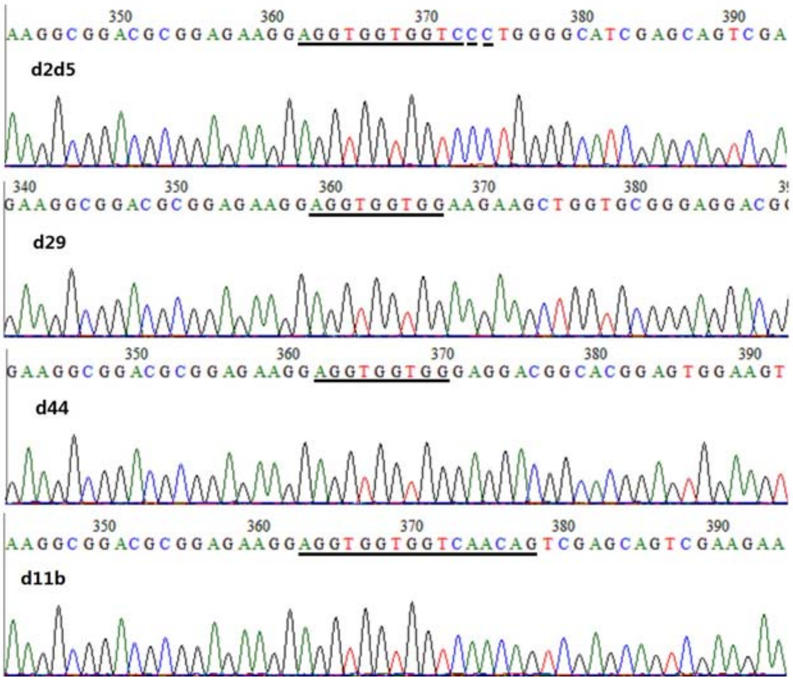

Supplemental Figure S1 Targeted mutations induced by CRISPR/Cas9 system at the *OsAOX1a* in T_0 generation of transgenic rice.

Mutated alleles shown were amplified from genomic DNA isolated from T_0 transgenic plants separately and sequenced after cloned into vectors. A, Sequence alignment of the target regions. The wild type sequence is shown at the top with the target sequence in blue and the PAM in orange. The number of clones representing each mutated alleles is shown in brackets. B, Sequencing chromatograms of exemplary mutations. Underlined showed the target sequence in corresponding mutated allele.

Supplemental Figure S2

A *OsAOX1b* site

Plant ID	Sequence	
	TTAGTTTAGTCACCGAGATGAGCTCCCGAATGGCCGGAGC	
Wild-type	TTAGTTTAGTCACCGAGATGAGCTCCCGAATGGCCGGAGC	
#1	TTAGTTTAGTCACCGAGATGAGCTC----GAATGGCCGGAGC	d2(x9)
	TTAGTTTAGTCACCGAGATGAGC--------GAATGGCCGGAGC	d4(x3)
#2	TTAGTTTAGTCACCGAGATGAGC------CGAATGGCCGGAGC	d3(x4)
	TTAGTTTAGTCACCGAGATGAGCTC-----------------------------...	d69(x4)
#3	TTAGTTTAGTCACCGAGATGAGCTCC--GAATGGCCGGAGC	d1(x6)
	TTAGTTTAGTCACCGAGATGAGCTC-----------------------------...	d31(x1)
#4	TTAGTTTAGTCACCGAGATGAGCTCC--GAATGGCCGGAGC	d1(x9)
	TTAGTTTAGTCACCGAGATGAGC--------GAATGGCCGGAGC	d4(x2)

B

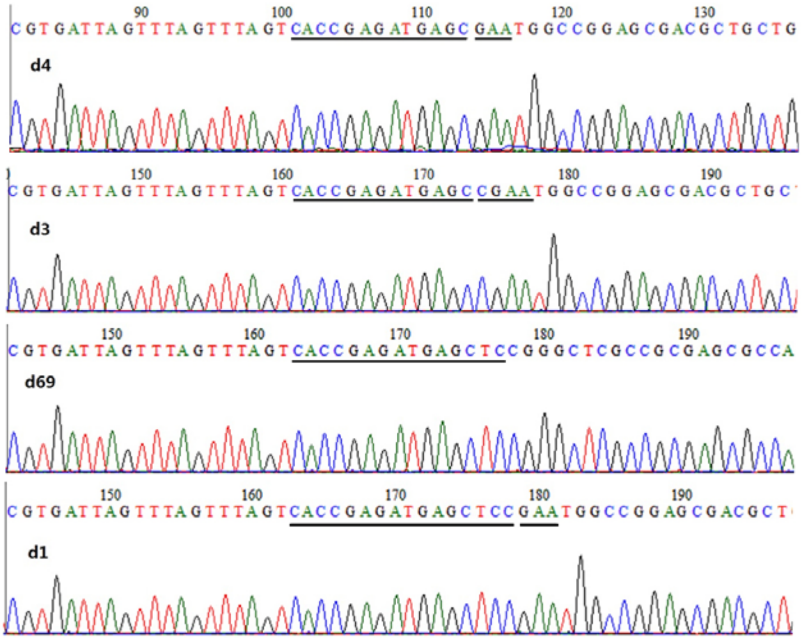

Supplemental Figure S2 Targeted mutations induced by CRISPR/Cas9 system at the *OsAOX1b* in T_0 generation of transgenic rice.

Mutated alleles shown were amplified from genomic DNA isolated from T_0 transgenic plants separately and sequenced after cloned into vectors. A, Sequence alignment of the target regions. The wild type sequence is shown at the top with the target sequence in blue and the PAM in orange. The number of clones representing each mutated alleles is shown in brackets. B, Sequencing chromatograms of exemplary mutations. Underlined showed the target sequence in corresponding mutated allele.

Supplementary Figure S3

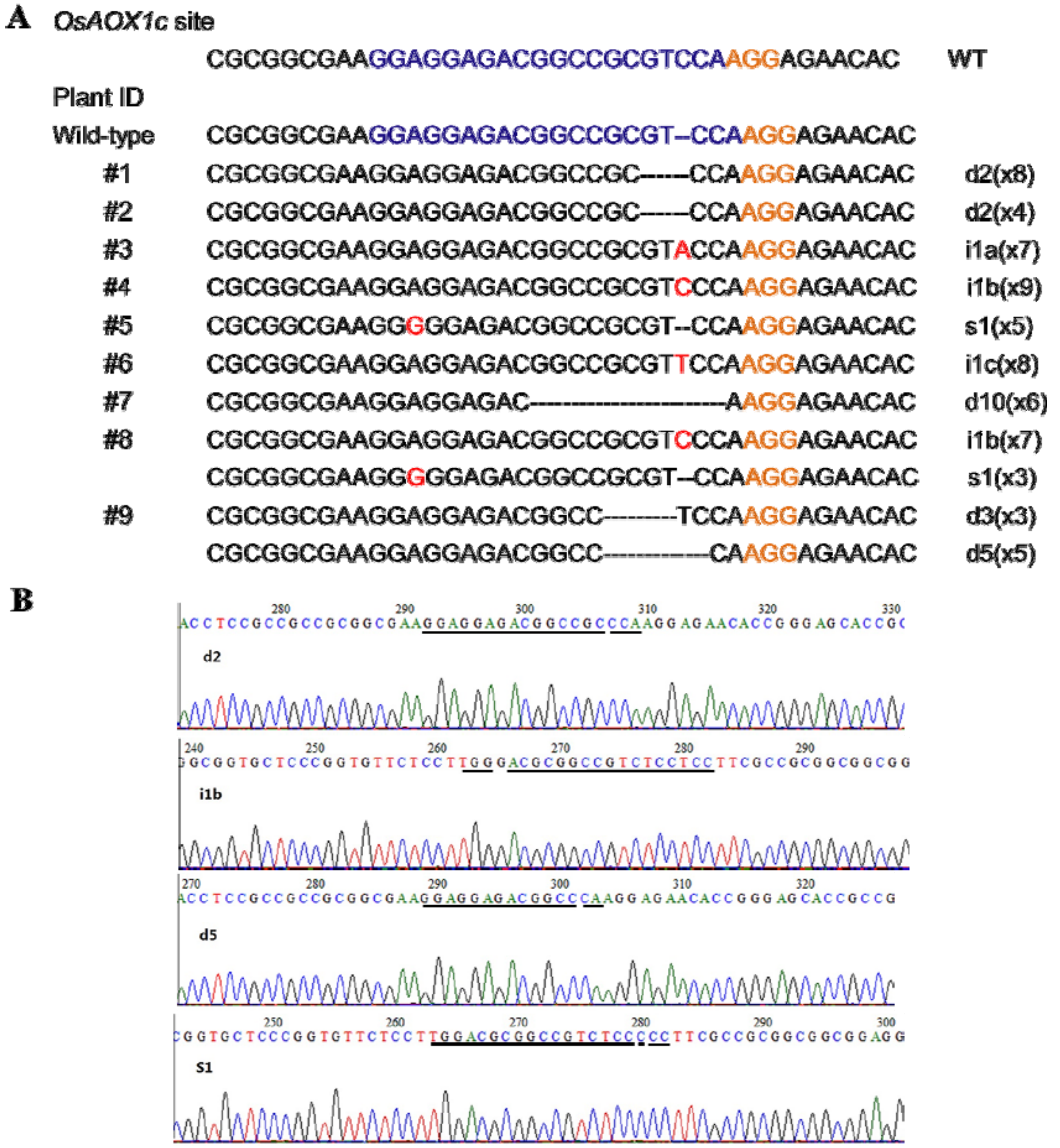

Supplemental Figure S3 Targeted mutations induced by CRISPR/Cas9 system at the *OsAOX1c* in T_0 generation of transgenic rice.

Mutated alleles shown were amplified from genomic DNA isolated from T_0 transgenic plants separately and sequenced after cloned into vectors. A, Sequence alignment of the target regions. The wild type sequence is shown at the top with the target sequence in blue and the PAM in orange. The insertion/replacement nucleotide was labeled as red. The number of clones representing each mutated alleles is shown in brackets. B, Sequencing chromatograms of exemplary mutations. Underlined showed the target sequence in corresponding mutated allele.

Supplemental Figure S4

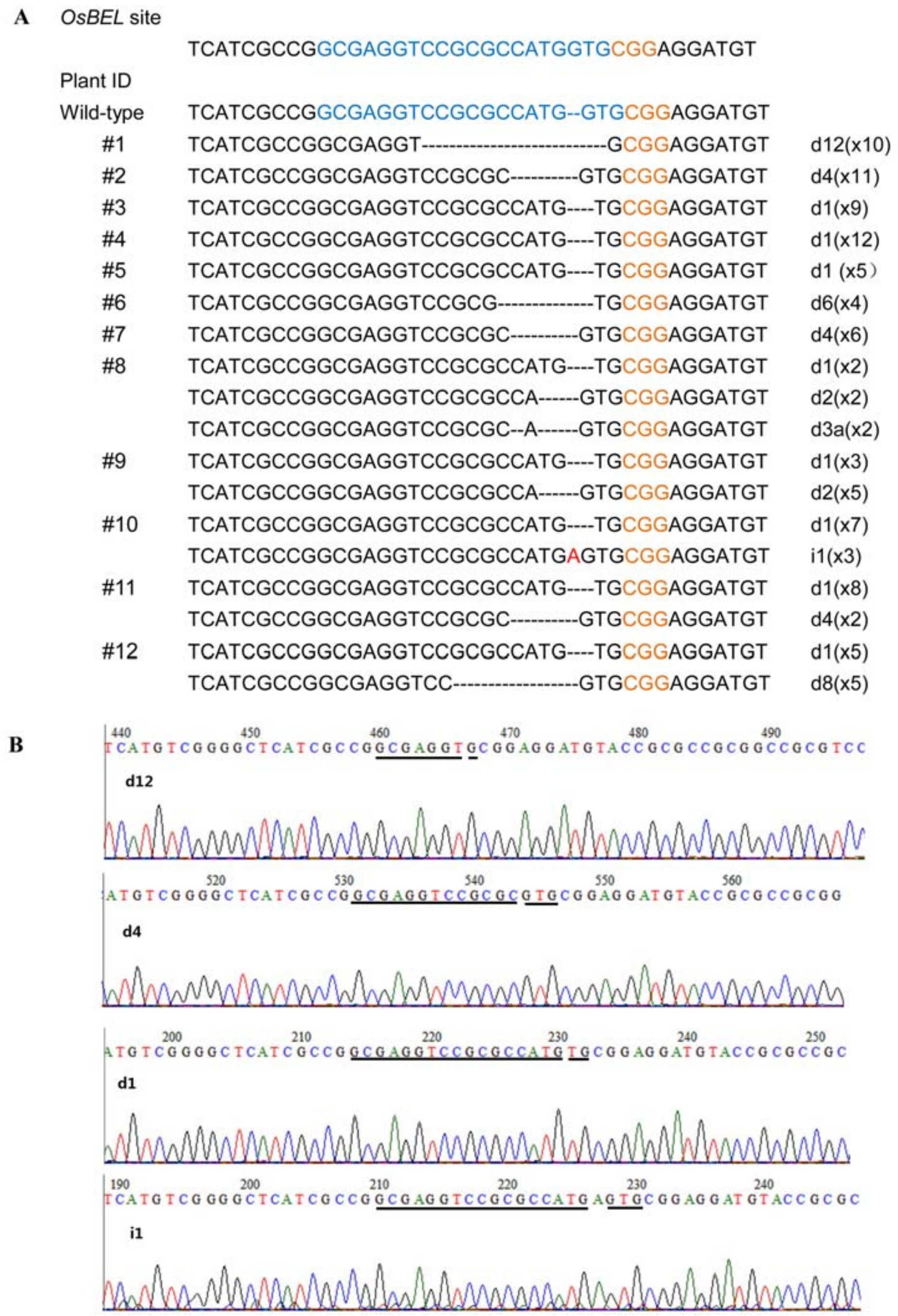

Supplemental Figure S4 Targeted mutations induced by CRISPR/Cas9 system at the *OsBEL* in T_0 generation of transgenic rice.

Mutated alleles shown were amplified from genomic DNA isolated from T_0 transgenic plants separately and sequenced after cloned into vectors. A, Sequence alignment of the target regions. The wild type sequence is shown at the top with the target sequence in blue and the PAM in orange. The insertion nucleotide was labeled as red. The number of clones representing each mutated alleles is shown in brackets. B, Sequencing chromatograms of exemplary mutations. Underlined showed the target sequence in corresponding mutated allele.

Supplemental Figure S5

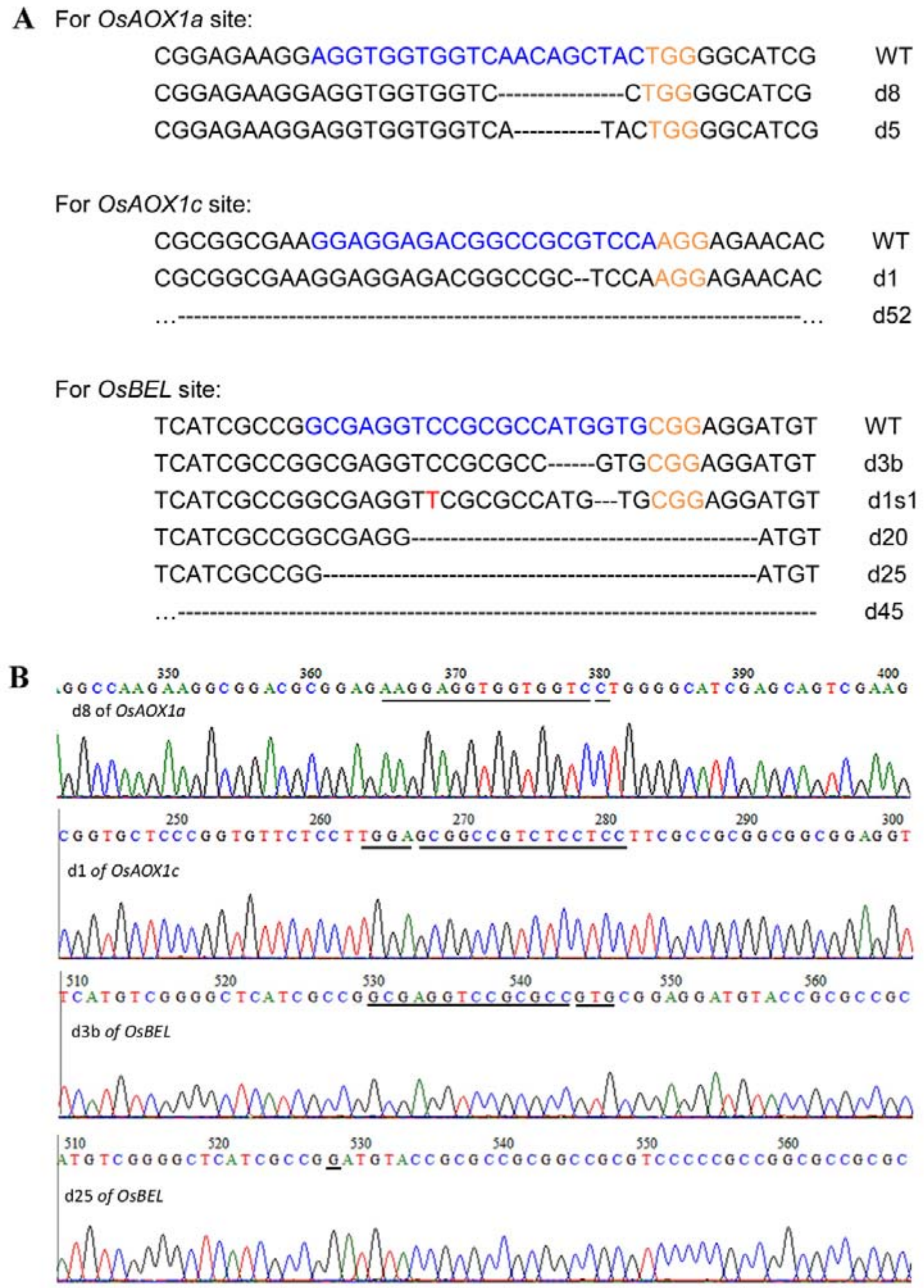

Supplemental Figure S5 New target mutations detected in the later generations of CRISPR/Cas9 transgenic plants.

Additional mutation types were observed from T_1 and T_2 generations of transgenic plants. A, Sequence alignment of the target regions. The wild type sequence around each target is shown at the top with the target sequence in blue and the PAM in orange. The replacement nucleotide was labeled as red. B, Sequencing chromatograms of exemplary mutations. Underlined showed the target sequence in corresponding mutated allele.

Supplemental Table S1 Detection different parts of T-DNA region in T_0 generations

Target Gene	Line#	Genotype	*HPT*	*Cas9*	*sgRNA*
OsAOX1a	1	d2d5,d11a	+	+	+
	2	d44,d29	+	+	+
	3	d11bd11b	+	+	+
	4	d3,d6	+	+	+
	5	WT	+	+	+
	6	WT	+	+	-
	7	WT	+	+	+
	8	WT	+	-	-
OsAOX1b	1	d2,d4	+	+	+
	2	d3,d69	+	+	+
	3	d1,d31	+	+	+
	4	d1,d4	+	+	+
	5	WT	+	+	+
	6	WT	+	+	+
	7	WT	+	+	-
OsAOX1c	1	d2d2	+	+	+
	2	d2d2	+	+	+
	3	i1ai1a	+	+	+
	4	i1bi1b	+	+	+
	5	s1,WT	+	+	+
	6	i1c,WT	+	+	+
	7	d10,WT	+	+	+
	8	i1b,s1	+	+	+
	9	d5,d3	+	+	+
	10	WT	+	-	+
	11	WT	+	+	-
	12	WT	+	+	+
OsBEL	1	d12d12	+	+	+
	2	d4d4	+	+	+
	3	d1d1	+	+	+
	4	d1d1	+	+	+
	5	d1,WT	+	+	+
	6	d6,WT	+	+	+
	7	d4,WT	+	+	+
	8	d1,d2,d3a,WT	+	+	+
	9	d1,d2,WT	+	+	+
	10	d1,i1	+	+	+
	11	d1,d4	+	+	+
	12	d1,d8	+	+	+
	13	WT	+	+	-
	14	WT	+	+	+

Supplemental Table S2. The primers used in this study

Target and PAM sequences used for gene editing in rice		
Target gene	Protospacer	PAM
OsAOX1a	AGGTGGTGGTCAACAGCTAC	TGG
OsAOX1b	CACCGAGATGAGCTCCCGAA	TGG
OsAOX1c	GGAGGAGACGGCCGCGTCCA	AGG
OsBEL	GCGAGGTCCGCGCCATGGTG	CGG
The primers used for check the transgenic plants		
Target gene	Genome check primer	
OsAOX1a	FP:5'-CGCAACCATCTCGTCAACAAA-3'	
	RP:5'- TAAAGCAAGACCACTTCCACTC-3'	
OsAOX1b	FP:5'- CTATCACAGCAACACAAGCCAA-3'	
	RP:5'- CCTAAAACTGAGCCACTTCCAT-3'	
OsAOX1c	FP:5'- AGCCACGAACAGAGCATCAACA-3'	
	RP:5'- AACAGCAAAAAAGAGAGGAAGC-3'	
OsBEL	FP:5'- CACCGAGCACGACGTGACCTTC-3'	
	RP:5'- CTTCCTCCTGACGCCGAACACG-3'	
The primers used for check the off-target mutations		
Putative off-target Sites	Off-target mutation check primers	
OsAOX1a-O1	FP: 5'-AGGTGGACCTGGATACGAAA-3'	
	RP: 5'-CACAATGACTAGGCTTATGAAA-3'	
OsAOX1a-O2	FP: 5'- TGCAGCTAGCGCAGCAACTA-3'	
	RP: 5'-GTTCGGAGTCTGGTTTTGAA-3'	
OsAOX1b-O3	FP: 5'-AATCTTCGTCGGGTCCATCTCC-3'	
	RP: 5'-GGTCCCCTACCGCCTTCCAC-3'	
OsAOX1b-O4	FP: 5'-CATGGTTGCTAAATCCAAAAT-3'	
	RP: 5'AAGCTGACTGACAGGGTAAA-3'	
OsAOX1c-O5	FP: 5'- GAAATCGCACGCACCTTGT-3'	
	RP: 5'- AATCCACCGCCGACGCTCC-3'	
OsAOX1c-O6	FP: 5'-CTCCTACCGCCGCATCTCGTG-3'	
	RP: 5'-GTGGTTGCCGTCCGGGTCAT-3'	
OsBEL-O7	FP: 5'- CATGTCGTTCGACGGCACC-3'	
	RP: 5'-CCTTGCTCTGGGCGATGGT-3'	
OsBEL-O8	FP: 5'-TGGCGTATGTTTATGTTCAGTTCC-3'	
	RP: 5'-ACCAATTCAATGGCTGTGGC-3'	
The primers used for check the presence of T-DNA fragments		
HPT	FP: 5'-CGCCGATGGTTTCTACAA-3'	
	RP: 5'-CGCCGATGGTTTCTACAA-3'	
OsCas9	FP: 5'-ATGGCCCCAAAGAAGAAGCGCA-3'	
	RP: 5'-TCAATCGCCGCCGAGTTGTGAG-3'	
sgRNA	FP: 5'-ATCTTGGAGGAATCAGATGT-3'	
	RP: 5'-TAACGGACTAGCCTTATTTT-3'	
